# YOLO SSPD: a small target cotton boll detection model during the boll-spitting period based on space-to-depth convolution

**DOI:** 10.3389/fpls.2024.1409194

**Published:** 2024-06-20

**Authors:** Mengli Zhang, Wei Chen, Pan Gao, Yongquan Li, Fei Tan, Yuan Zhang, Shiwei Ruan, Peng Xing, Li Guo

**Affiliations:** ^1^ College of Information Science and Technology, Shihezi University, Shihezi, China; ^2^ School of Information Science and Engineering, Xinjiang College of Science and Technology, Korla, China

**Keywords:** cotton boll detection, cotton yield estimation, transfer learning, YOLOv8, UAV

## Abstract

**Introduction:**

Cotton yield estimation is crucial in the agricultural process, where the accuracy of boll detection during the flocculation period significantly influences yield estimations in cotton fields. Unmanned Aerial Vehicles (UAVs) are frequently employed for plant detection and counting due to their cost-effectiveness and adaptability.

**Methods:**

Addressing the challenges of small target cotton bolls and low resolution of UAVs, this paper introduces a method based on the YOLO v8 framework for transfer learning, named YOLO small-scale pyramid depth-aware detection (SSPD). The method combines space-to-depth and non-strided convolution (SPD-Conv) and a small target detector head, and also integrates a simple, parameter-free attentional mechanism (SimAM) that significantly improves target boll detection accuracy.

**Results:**

The YOLO SSPD achieved a boll detection accuracy of 0.874 on UAV-scale imagery. It also recorded a coefficient of determination (R^2^) of 0.86, with a root mean square error (RMSE) of 12.38 and a relative root mean square error (RRMSE) of 11.19% for boll counts.

**Discussion:**

The findings indicate that YOLO SSPD can significantly improve the accuracy of cotton boll detection on UAV imagery, thereby supporting the cotton production process. This method offers a robust solution for high-precision cotton monitoring, enhancing the reliability of cotton yield estimates.

## 1 Introduction

Cotton yield estimation is essential for the cotton production process and can influence the price trend in the cotton market (Sarkar et al., 2023). Cotton yield estimation can be carried out by boll detection during the cotton fluffing period (Pokhrel et al., 2023; Torgbor et al., 2023). The quantity of cotton bolls directly affects the cotton harvest and is also a key indicator for assessing the quality of cotton (Shi et al., 2022). A high precision boll number detection model can quickly and accurately model yield estimation before harvesting and make planting management related decisions, which is economically vital for cotton production (Thorp et al., 2020; Naderi Mahdei et al., 2023).

Traditional cotton production information detection methods require sampling and frequent manual observation of cotton fields (Tian et al., 2022; Kurihara et al., 2023). With the continuous improvement of land transfer rate, large-scale planting rate and technological content, driven by the whole mechanization, many new technologies have been applied to the field of cotton production, improving the development of cotton production process intelligence (Muruganantham et al., 2022; Yan et al., 2022). Although high-resolution, ground-shot images are not suitable for cotton boll detection in field environments due to their high acquisition costs. As remote sensing technology develops, satellite positioning system and geographic information system (GIS), unmanned aerial vehicle (UAV) remote sensing technology has found broad applications (Dhaliwal and Williams, 2023; Hu et al., 2023; Kumar et al., 2023; Priyatikanto et al., 2023). Due to the small scale of cotton bolls and the complexity of the field background, large-scale monitoring methods such as satellite remote sensing cannot describe the detailed changes of cotton bolls in a small-scale range. Low-altitude UAV remote sensing acquires the benefit of short cycle time and fast speed, so it provides technical support for small- and medium-scale crop growth monitoring (Eskandari et al., 2020; Fernandez-Gallego et al., 2020; Hassanzadeh et al., 2021; Palacios et al., 2023).

UAVs provide excellent image acquisition flexibility at flight altitude, flight area and various weather conditions for fast and accurate crop monitoring (Bouras et al., 2023; X. Wang, Lei, et al., 2023). UAV remote sensing combined with machine learning algorithms is an essential area of re-search in target detection studies based on UAV remote sensing images. In the study of automated agave detection, the utilization of UAV image data has demonstrated remarkable accuracy (Flores et al., 2021). Moreover, red, green, blue (RGB) aerial imagery from UAV, coupled with the faster regions with convolutional neural network (Faster R-CNN) object detection model, prove effective in estimating plant density (Velumani et al., 2021). The application of UAV image data for training convolutional neural networks (CNNs) shows superior performance compared to traditional machine learning methods (Impollonia et al., 2022; Amarasingam et al., 2024; Skobalski et al., 2024; Zou et al., 2024). High-resolution images significantly enhance the accuracy of target detection. Collection of high-resolution UAV RGB images provides a methodology for counting plants at different growth stages of sunflowers and corn seedlings (Bai et al., 2022). High-resolution UAV images, when combined with suitable image segmentation algorithms, serve as a basis for detection counting and analysis. In a study focused on the detection and counting of citrus trees using high-resolution UAV images, the connected component labelling (CCL) algorithm was employed to segment and label individual citrus trees in images (Donmez et al., 2021). The relationship between image based manual counting and algorithmic counting demonstrates high precision and efficiency through the utilization of frequency filters, segmentation, and feature extraction techniques (Azizi et al., 2024; Liu et al., 2024). Given sufficient data, pre-trained deep learning models offer enhanced generalization performance in target detection tasks. The pre-trained ResNet 17 model, when applied to UAV-captured RGB images of cotton seedlings, enables real-time estimation of the quantity and canopy size of the seedlings in each frame (Feng et al., 2020). Building on the success of this method, researchers have further integrated transfer learning techniques into a new framework that combines remote sensing and deep learning to enhance processing efficiency. This integrated framework has proven particularly effective in sparse counting tasks for different plant species, such as potatoes and lettuce (Machefer et al., 2020). Estimating crop density using vegetation indices is applicable in the early and middle stages of crop growth, but its performance is limited at maturity due to the gradual onset of plant senescence, wilting leaves, and the impact of crop fruits (Huang et al., 2018).

Following the analysis of various multispectral and RGB vegetation indices, a neural network model can integrate the analytical results to estimate vegetation coverage and crop density (García-Martínez et al., 2020). Remote sensing imagery has been widely employed for crop segmentation in the later stages of crop growth, yielding significant results. UAV images are also utilized in computing the elevation difference between the crop canopy and exposed soil surface, extracting cotton height during the boll spitting period, and using it as a basis for estimating cotton yield. The specific process involves validating UAV-based height measurements using known ground reference points, segmenting crop rows, and obtaining a plant height map based on global positioning system (GPS) and image features (Feng et al., 2019). Remote sensing image segmentation can be employed to detect the quantity of target cotton bolls since cotton often exhibits distinct optical features (such as color and morphology) from branches and leaves in the later stages of growth. A cotton boll threshold segmentation detection algorithm based on UAV remote sensing images is proposed. Initially, spectral thresholds are derived from input images through adaptive applications, automatically distinguishing cotton bolls from other non-target items. Subsequently, the derived thresholds and other morphological filters are utilized for binary cotton boll classification to reduce result noise (Yeom et al., 2018). Combining UAV remote sensing data with multispectral images and cotton boll pixel coverage enables the construction of a high precision cotton boll detection model. This model primarily utilizes a Bayesian regularized back propagation (BP) neural network to predict cotton yield from the quantity of cotton bolls and spectral indices(R. Xu et al., 2018; W. Xu et al., 2021).

Due to the extension and interlacing of cotton leaves in the background of the cotton field and the complex changes in the external environment, the morphological characteristics of cotton bolls in the field are variable and overlapping. Therefore, for cotton boll detection in a field environment, the boll-spitting period is considered the ideal phase. However, due to the large number and small size of cotton bolls, a specific detection model is required to achieve accurate applications (Fue et al., 2018). The YOLO series has undergone multiple updates and iterations, making it suitable for detection and segmentation in agriculture. This model builds upon the YOLOv8 architecture with added modules for feature processing, significantly improving the detection accuracy of small objects in UAV images (G. Wang, Chen, et al., 2023). The real-time YOLOv8 model has been effectively applied for detecting kiwifruit diseases, providing real-time disease estimates (Xiang et al., 2023). Additionally, to address the challenge of strawberry ripeness detection, the YOLOv8s model and the LW-Swin Transformer module have been employed to support the strawberry picking process in orchard management (Yang et al., 2023).

**Figure 1:**
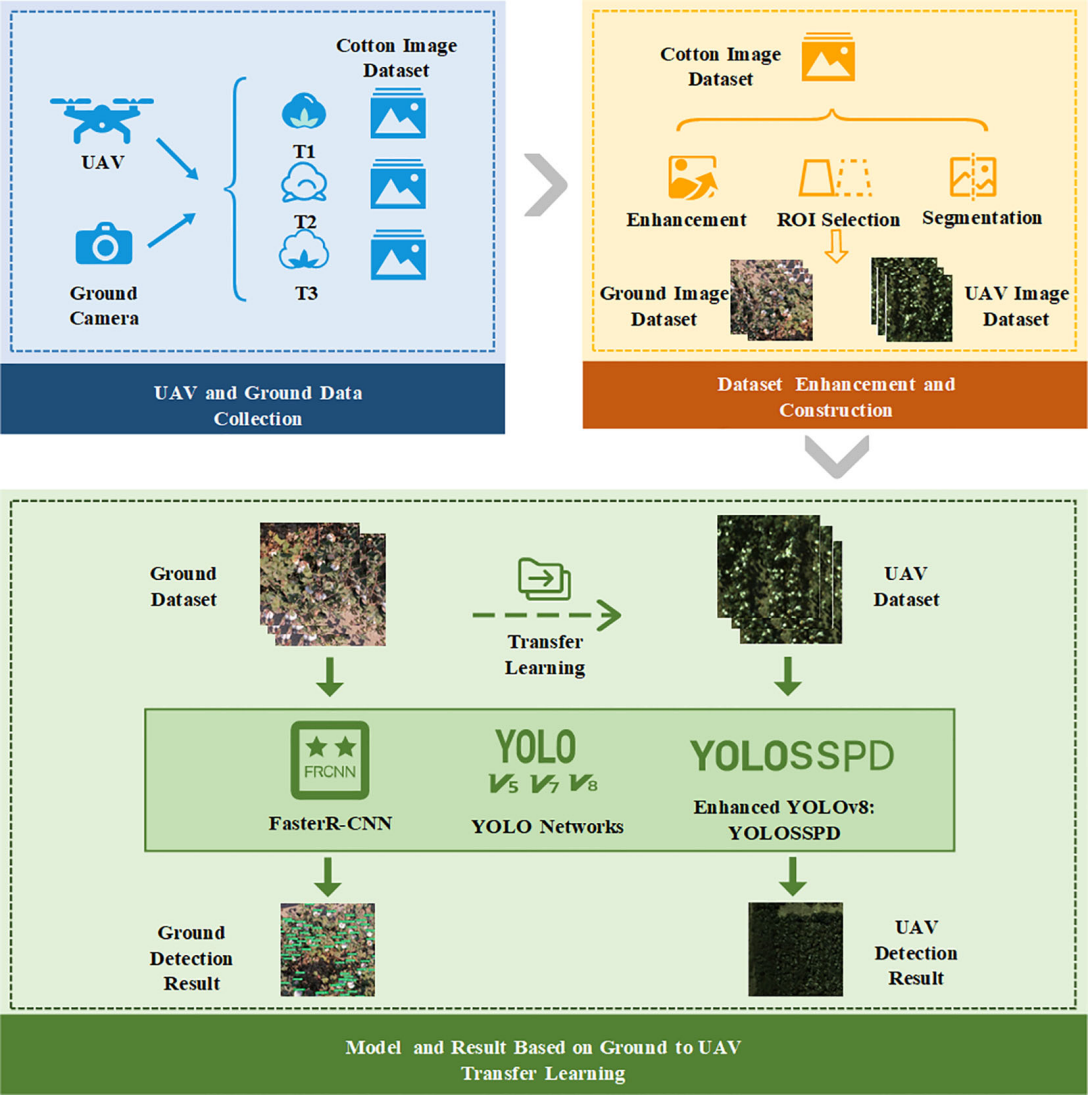
The abstract process framework of this study.

This study introduces an enhanced detection model, YOLO small-scale pyramid depth-aware detection (SSPD), based on YOLOv8 to improve the accuracy of UAV-based cotton boll detection during the boll spitting period. High-resolution ground cotton boll images were initially captured and utilized to train data on network models such as YOLO SSPD. The trained model was subsequently transferred to UAV remote sensing images for the detection and counting of cotton bolls. The Detailed Process Overview is Shown in **Figure 1**.

## 2 Materials and methods

### 2.1 Dataset acquisition and preprocessing

**Figure 2:**
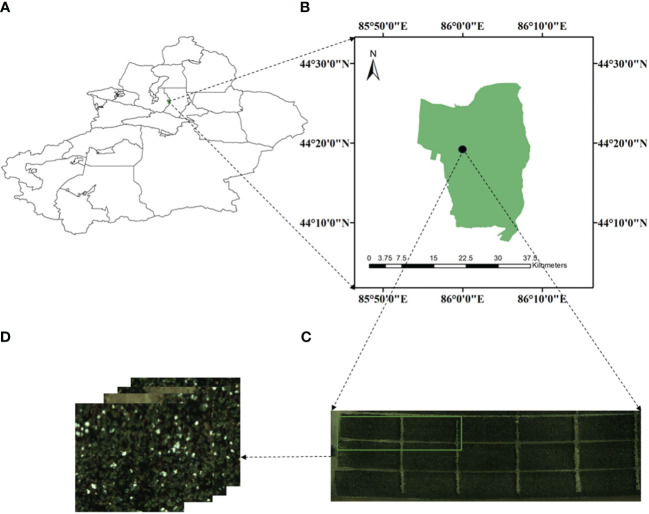
Overview of study area: **(A)** illustrates the graph of Xinjiang, **(B)** represents the area of Shihezi, **(C)** represents the testing region, Cotton boll image acquisition experimental area, the photos in **(D)** are the RGB images taken by a drone.

This research was carried out from August to October 2021 in the Second Company of Experimental Field of Xinjiang Shihezi University (latitude 44°18′N, longitude 85°58′E, average altitude 443 m), as shown in **Figure 2**. The experimental area was planted with “Xinlu Early No. 53” and “Xinlu Early No. 74”, utilizing the planting pattern “one film, three cylinders and six rows” with the design of a comprehensive and cramped row. The chosen cotton variety was “Xinlu Early No. 53”, and the planting density is 20 plants per square meter. The image data collection activities were carried out in three stages of the cotton fluffing period. The three stages of filming were 5 days after the first defoliant spraying (T1, September 8th), 3 days after the second defoliant spraying (T2, September 15th) and 7 days before cotton picking (T3, September 25th).

### 2.2 UAV image data acquisition and processing

**Figure 3:**
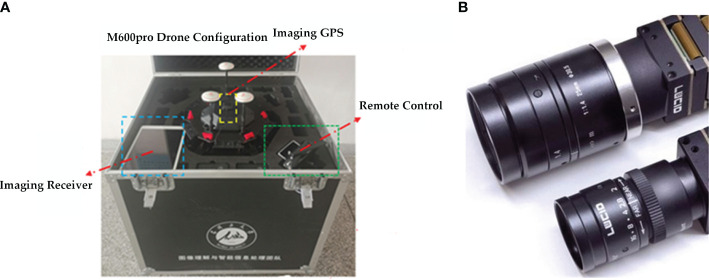
The DJI drone that collected the data, where **(A)** is the configuration of the DJI M600pro drone and **(B)** the RGB camera carried by the drone.

This study uses a DJI M Atrice M600 PRO UAV (Shenzhen, China) with third-party hardware and software extensions, a global positioning system (GPS) positioning system, a flight imaging receiver, an a3 Pro flight controller, a Lightbridge 2 high definition (HD) digital mapper, and a remote control, with a load capacity of 6.0 kg and an Isuzu Optics real-time camera (Hsinchu County, Taiwan, China). The UAV captured datasets were all RGB images, and the real-time camera parameters are shown in **Table 1**. Each time the images were taken, three altitudes were flown, 60 meters, 40 meters and 20 meters from the ground. The UAV flight speed was 2.8 m/s, the camera was oriented parallel to the main flight path, the heading overlap rate was 70%, the side overlap rate was 60%, the gimbal pitch angle was -80°, and the camera mode was set to isometric intervals to increase the efficiency of the shooting as well as to obtain a clear image of the UAV. The camera configured and carried by the UAV is shown in **Figure 3**.

**Table 1:** Configuration of the hyperspectral camera carried by the drone.

Parameter	Value
Spectral bandwidth	<15nm,collimated
Base imager type	CMOS^1^ imager, CMOSIS CMV^2^ 2000based
Spatial resolution	408*216 per band
Frame rate	Up to 340 hyperspectral cubes/second
Pixel pitch	5.5μm
Bit depth	7or10bit
RGB pixel	4 million

^1^CMOS-complementary metal-oxide semiconductor. ^2^CMV-CMOSIS machine vision.

**Figure 4:**
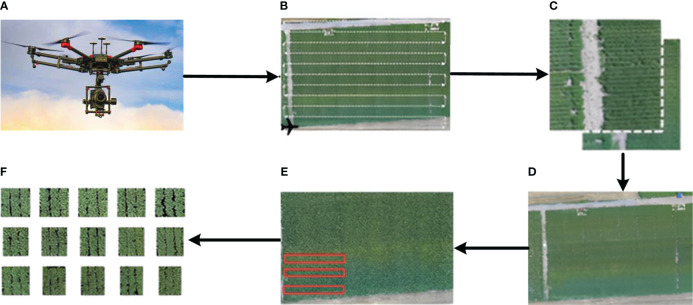
Remote sensing image processing flow: **(A)** UAV commissioning, **(B)** UAV flight parameter setting, **(C)** raw image acquisition, **(D)** remote sensing imaging stitching, **(E)** ROI selection and **(F)** datasets cropping.

Pictures taken by UAVs are characterized by small image size, large data volume, and rich spatial information. Still, environmental factors also directly affect, such as sunshine, wind direction, etc. Therefore, even if multiple pictures are acquired in the same environment, there will be differences in sensitivity and color, which will directly affect the accuracy of the subsequent detection of feature points, thus directly affecting the final use of remote sensing data from UAVs for target detection using UAV remote sensing data. In this paper, the steps of UAV remote sensing image processing include UAV flight parameter setting, raw image acquisition, remote sensing imaging stitching, region of interest (ROI) selection and datasets cropping, and the remote sensing image processing steps are shown in **Figure 4**.

### 2.3 Datasets enhancement and construction

The image annotation tool LabelImg (free and open source, Taiwan, China) was installed, and each cotton bolls were annotated. An extensible markup language (XML) record file was generated for the training images output from each boll for better image data management and analysis in subsequent studies. In this study, the entirety of six training datasets was prepared, including 600 images of each of T1, T2 and T3 randomly selected from the ground data set and 50 segmented images of each of T1, T2 and T3 irrelevantly chosen from the UAV images. The training images were randomly cropped from the UAV RGB composite images, each with a size of 640 x 640 pixels. Ground images of 7,000, 7,500, and 6,000 were acquired for the three periods, and UAV cropped images of 250, 450, and 800 were acquired for the three flight altitudes. The above two different scales of images were randomly assigned in the proportion of 3:1:1 for the training, validation and testing of the cotton bolls detection model.

**Figure 5:**
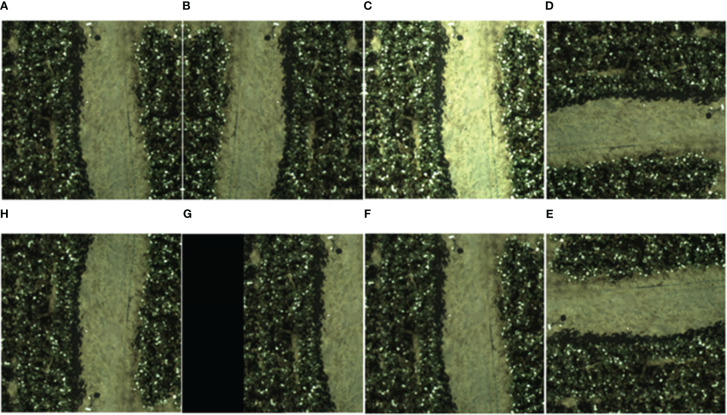
UAV expanded RGB image datasets methods: **(A)** original image, **(B)** horizontal mirroring, **(C)** increasing brightness, **(D)** rotating 90° to the right, **(E)** vertical mirroring, **(F)**image panning, **(G)** increasing noise, and **(H)** rotating 90 to the left.

During the construction of the cotton bolls datasets, due to the direct influence of various reasons such as shooting time, climate, flight speed, camera viewpoint, etc. The cotton boll image data varied greatly, resulting in data imbalance, so it is necessary to carry out data enhancement on the cotton bolls image datasets. To further enhance the quality of the datasets, methods, for example, image rotation, image panning, image mirroring and adding image noise, are used to perform data enhancement on the existing datasets. The way the UAV enhanced the RGB image data is shown in **Figure 5**.

### 2.4 Cotton boll detection models

The models were trained on a platform equipped with an NVIDIA GeForce RTX 3060 laptop graphics processing unit (GPU) with 16GB of random-access memory (RAM). This setup provides powerful graphics processing, which is critical for handling complex computations in deep learning models. The system runs on Windows 10 x64 with a 12th generation Intel^®^ Core™ i5–12500H central processing unit (CPU), which supports efficient multitasking and fast data processing. In addition, the device features 1.0TB of storage capacity, allowing for extensive data processing and model training without storage limitations. The Pytorch framework version used is 1.7.1, which is known for its flexibility and efficiency in model development. Optimized computational performance with compute unified device architecture (CUDA) 11.0 and CUDA deep neural network (cuDNN) 8.0.5 ensures faster training times and enhanced reproducibility of results.

#### 2.4.1 Faster R-CNN

Faster R-CNN (https://github.com/jwyang/faster-rcnn.pytorch) (Mai et al., 2020) is an improved version of fast regions with convolutional neural network (Fast R-CNN) that draws features straight from the original input image. It then uses ROI Pooling to extract feature vectors of a specific length for each ROI on the feature map of the whole image. It regresses the feature vectors directly on them using multiple full convolution (FC) layers. Two FC branches are then used to predict the ROI-related categories and boxes separately, which significantly improving speed and prediction. The first part of the network architecture uses convolution layer stacking to extract the feature map from the image, then fixes the data dimensions using region pooling. The Region Proposal Network (RPN) network is the second part, which mainly serves to generate alternate regions. The third part of ROI Pooling is primarily responsible for the feature maps of the convolutional network inputs, and the exact proposals generated by the RPN training (Duan et al., 2019; Chen et al., 2020; Zhang et al., 2021), and the pooling process is used to implement edge regression and region classification. In this study, the image input size is set to 640 × 640, the learning rate is configured to 0.001, the step size is adjusted to 5, the batch size is fixed at 16, and the number of iteration rounds is 500.

#### 2.4.2 YOLOv5

On the input side of YOLOv5 (https://github.com/ultralytics/yolov5), the mosaic data information boost technique replaces the traditional single-cut mix data information enhancement method of the previous generations. It employs the self-fitting stroke frame method and self-fitting image compression (Ghiasi et al., 2021). Cross stage partial (CSP) and focus structures are introduced in the Backbone part of the network to expand the input channels for subsequent slicing operations. The neck part of the network greatly improves the deep learning capability of the network by combining feature pyramid networks (FPN) and path aggregation network (PAN), and applies PAN to the three effective feature layers for better fusion of features from different layers. In addition, in order to obtain more accurate output results, the neck also adopts generalized intersection over union (GIOU) loss as the loss function for edge regression to achieve more efficient model analysis. In this study, the image input size is 640×640, because it is cotton boll single target detection, the output category of the network, nb_classes, is changed to 1, the training weights are yolov5s, the optimizer chosen is stochastic gradient descent (SGD), the batch size is 16, the iteration rounds epoch is 500, and the learning rate is set as 0.001, and the rest are default settings.

#### 2.4.3 YOLOv7

YOLOv7 (https://github.com/WongKinYiu/yolov7) inherits the architecture of YOLOv5, including the configuration information settings, training process, inference and testing procedures. Additionally, YOLOv7 adopts the structure and methods of hyperparameter tuning and implicit knowledge learning from YOLOR. It also incorporates YOLOX’s Optimal Transport Assignment (OTA) strategy for positive sample matching strategy. YOLOv7 itself also features an efficient aggregation network, reparametrized convolution, extra training module and model scaling (C.-Y. Wang, Bochkovskiy, and Liao 2023). Among these, the efficient aggregation network enhances the learning efficiency and aggregation ability of the network system by controlling the shortest and longest gradient paths (Zhao et al., 2023). The auxiliary training method and deep supervision in the YOLOv7 model add additional neurons to the network system to enhance the model’s accuracy. Notably, the auxiliary training method is only employed during the training process and does not degrade the accuracy of the model validation and testing (Jiang et al., 2022). In this study, the parameters are set as follows, the pre-training weight is YOLOv7-tiny, the optimizer is Adam, the batch size is 8, and the epoch is 500.

#### 2.4.4 YOLOv8

**Figure 6:**
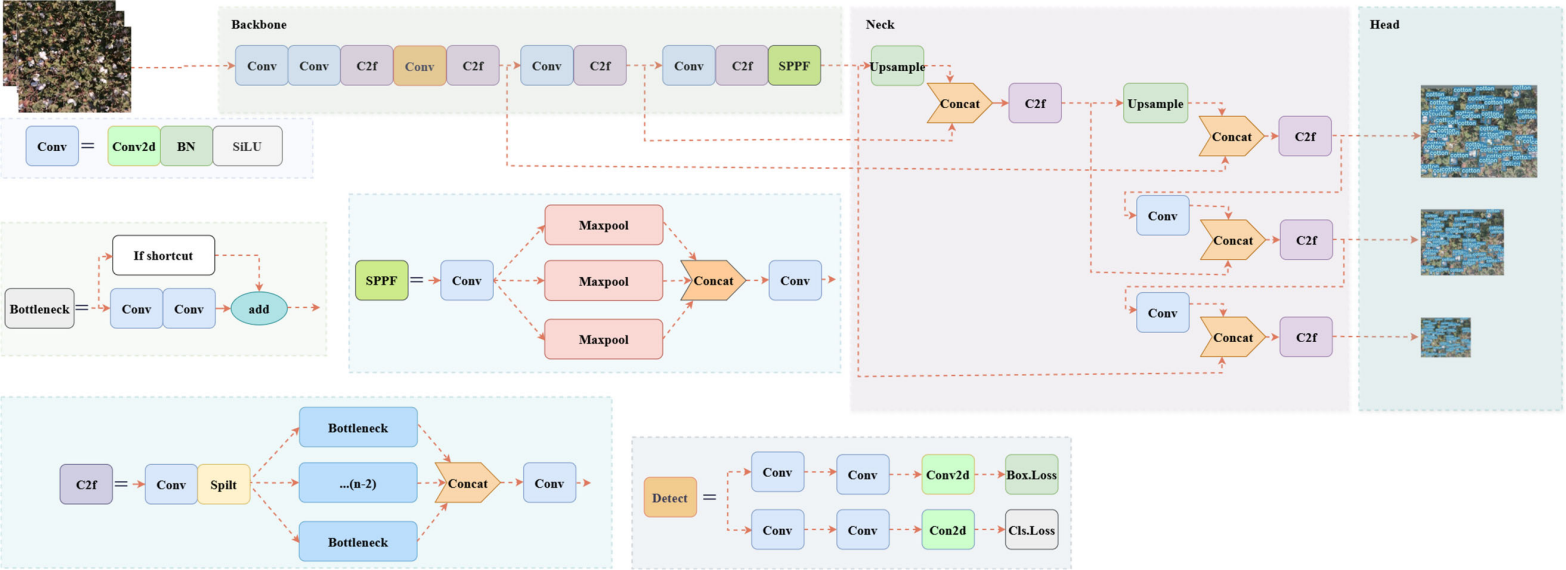
YOLOv8 model structure.

YOLOv8 (https://github.com/ultralytics/ultralytics) represents the latest advancement in the YOLO series of object detection models, showcasing superior performance in terms of both speed and accuracy compared to its predecessors. Building upon the foundation of earlier versions, YOLOv8 introduces notable enhancements. In the backbone architecture, YOLOv8 refines the C3 structure of YOLOv5 to the C2f structure. The C2f modification not only preserves the lightweight nature but also facilitates the acquisition of more informative features during the gradient descent process. Within the head component, YOLOv8 transitions from a coupled head to a decoupled head, departing from the anchor box structure employed in prior iterations in favor of an Anchor-Free approach. Moreover, YOLOv8 incorporates an outstanding dynamic allocation strategy in the design of its loss function. This strategic approach enhances the adaptability of the model during training. Notably, YOLOv8 demonstrates versatility by extending its applicability to earlier versions of the YOLO series, delivering commendable performance across image detection, segmentation, and classification tasks. The structure of Yolov8 is shown in **Figure 6**.

#### 2.4.5 YOLO SSPD

YOLO SSPD is designed based on the YOLOv8 architecture to address the challenges of small and dense cotton boll targets and complex field backgrounds in UAV-scale scenarios. SPD-Conv (https://github.com/LabSAINT/SPD-Conv) is a combination of space-to-depth layer and non-strided convolution. To mitigate the loss of image information during network propagation, the SPD-Conv structure is introduced (Sunkara and Luo, 2022). Equations 1–3 elucidate the principles of SPD convolution. The input feature map *X* with dimensions *S*×*S*× $C_{1}$ . The SPD transformation downsamples *X* using a scale parameter scale . For each position (*i*, *j*) in *X*, *X* is sliced into $scale^{2}$ sub-feature maps $f_{x,y}$ , where *x*, *y*∈ {0, 1, …, scale−1}. The sub-feature maps are extracted as follows:


(1) $$f_{x,y}=X\left[x:S:scale,y:S:scale\right]$$

Each sub-feature map $f_{x,y}$ downsamples *X* by extracting pixels at intervals of scale , and the dimensions of each $f_{x,y}$ are $\left(\frac{S}{\text{scale}},\frac{S}{\text{scale}},C_{1}\right).$ These sub-feature maps are then concatenated along the channel dimension to form a new feature map *X*′:


(2) $$X'\;=\;concatenate\left(\left\{f_{x,y}|x,y\in \left\{0,1,\ldots ,scale-1\right\}\right\},axis=channel\right)$$

The main purpose of this transformation is to increase the channel dimension while reducing the spatial dimensions of the feature map. The dimensions of the new feature map *X*′ are $\left(\frac{S}{\text{scale}},\frac{S}{\text{scale}},scale^{2}\times C_{1}\right)$ . A non-strided (stride=1) convolution operation is applied to *X*′ using *C*2 filters. This convolution transforms *X*′ into *X*′′ as follows:


(3) $$X=Convolution\left(X',filters=C_{2},stride=1\right)$$

**Figure 7:**
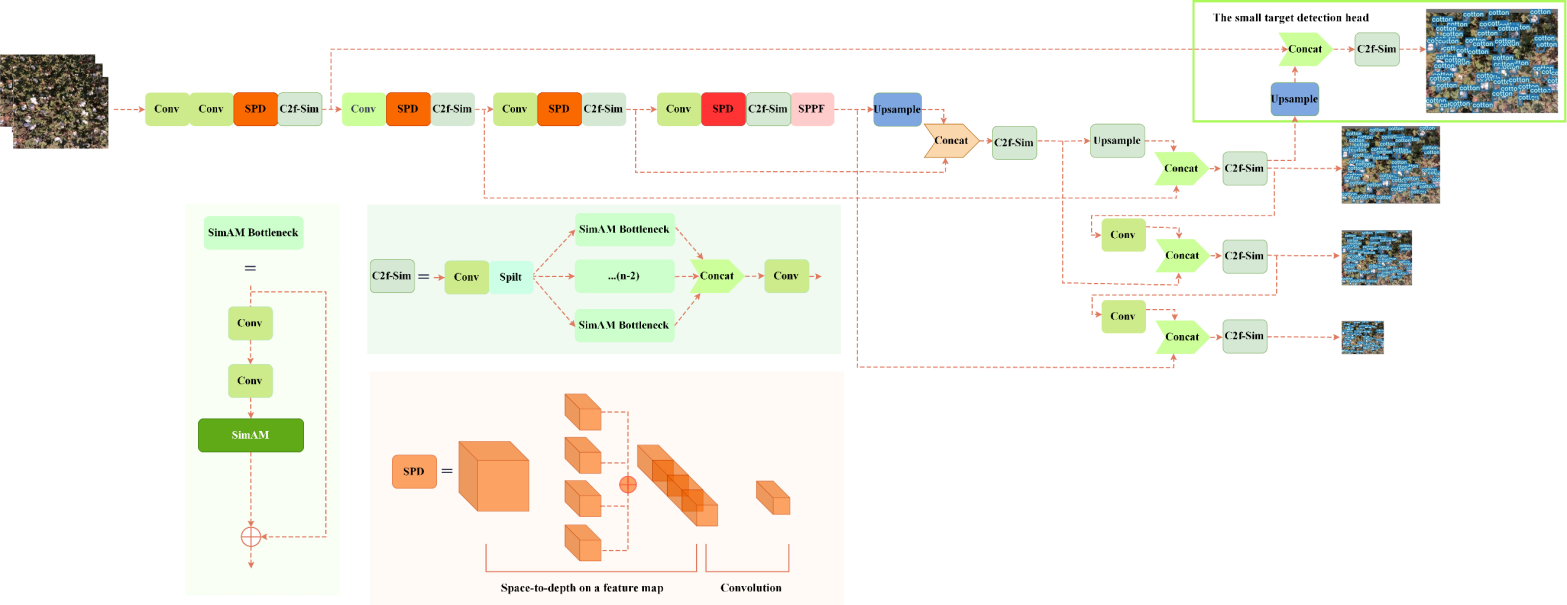
YOLO SSPD model structure.

This convolution operation aims to retain as much discriminative feature information as possible, preventing the loss of information. The dimensions of the output feature map *X*′′ are: $\left(\frac{S}{\text{scale}},\frac{S}{\text{scale}},C_{2}\right)$ . By scaling the image proportion before inputting it into the detection network, the space-to-depth layer preserves channel dimension information throughout the feature mapping process, effectively preventing information loss (Wan et al., 2024). Additionally, non-strided convolutions are added after the space-to-depth layer to expedite image processing. The simple parameter-free attention mechanism (SimAM), while not increasing computational parameters, serves as a versatile attention mechanism, enhancing model performance. When dealing with UAV images, this not only accelerates computation speed but also improves overall model efficiency. The small target detection head finds widespread applications in the industry, addressing challenges related to inconspicuous features and potential information loss during training, thereby enhancing detection capabilities. Integrating the small target detection head into YOLO SSPD contributes to improved accuracy in identifying small target cotton bolls. **Figure 7** illustrates the network structure of the YOLO SSPD.

### 2.5 Transfer learning based cotton boll detection from UAV RGB images

Transfer learning involves improving performance in a newly acquired task by leveraging knowledge gained from a closely related task that has already been mastered. To address the issue of limited training instances and low resolution of UAV remote sensing images, we first train the model on ground boll image data. Then, the trained model is applied to the boll recognition and detection task on UAV RGB images. Image size, quantity and quality are essential factors affecting the setting of training parameters, and in order to achieve the best training effect, these parameters must be refined to improve further the correctness and credibility of modelling (Tedesco-Oliveira et al., 2020; Park and Yu, 2021). In this study, the transfer learning model is configured with a learning rate of 0.0005, a batch size of 8, and a total of 500 iteration rounds.

### 2.6 Evaluation indicators

In this paper, single target detection of cotton bolls was investigated, so the model evaluation metrics selected included precision, recall, F1 score, average precision, average precision (AP) for a single class, and coefficient of determination (R^2^), relative root mean square error (RMSE) and root mean square error (RRMSE), which were calculated using the formulas shown below. Equations 4–10 are introduced as metrics for subsequent model performance evaluation.


(4) $$Precision=\frac{TP}{TP+FP}$$

(5) $$Recall=\frac{TP}{TP+FN}$$

(6) $$F1\;=\;2\times \frac{Precision\times Recall}{Precision+recall}$$

(7) $$AP=\int_{0}^{1}P\left(r\right)dr$$

(8) $$R^{2}=1-\frac{\sum_{1}^{n}{\left(p_{i}-c_{i}\right)}^{2}}{\sum_{1}^{n}{\left(p_{i}-{\bar{p}}_{i}\right)}^{2}}$$

(9) $$RMSE=\sqrt{\frac{\sum_{1}^{n}{\left(p_{i}-c_{i}\right)}^{2}}{n}}$$

(10) $$RRMSE=\frac{\sqrt[{n}]{\frac{1}{n}\sum_{1}^{n}\left(p_{i}-c_{i}\right)}}{\sum_{1}^{n}p_{i}}\times 100\%$$

Where True positive ( TP ) represents correct prediction of cotton bolls, False positive ( FP ) represents misidentification of background noise as cotton bolls, and False negative ( FN ) represents misidentification of cotton bolls as background noise. The value range of Precision and Recall is between 0 and 1, so the value range of AP is also in the range of [0,1]. $p_{i}$ , ${\bar{p}}_{i}$ and $c_{i}$ are the quantity of manually labelled bolls in the i -th image, the mean of the amount of manually labelled bolls in the i -th image and the count of bolls obtained by prediction, correspondingly. n is the total of test images.

## 3 Results

### 3.1 Results of ground cotton boll detection models

**Figure 8:**
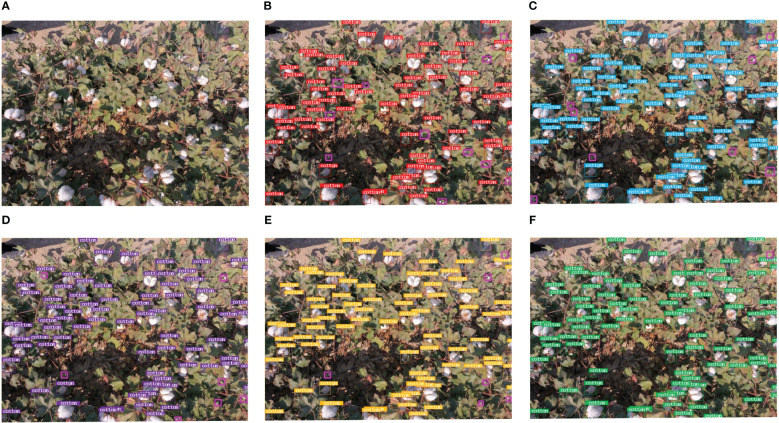
The model detection results (Pinkish-purple boxes show missed bolls): **(A)** Original image, **(B)** Faster R-CNN, **(C)** YOLOv5, **(D)** YOLOv7, **(E)** YOLOv8, **(F)** YOLO SSPD.

**Table 2** displays the outcomes of cotton boll recognition and detection in ground image data at different time intervals utilizing various object detection networks. When employing models like Faster R-CNN, a consistent performance trend is observed across different time periods, with T2 > T1 > T3. This phenomenon is attributed to the suboptimal effect of defoliant spraying during the T1 period. In the T3 period, when cotton flowers are fully open, distinguishing targets becomes challenging, resulting in instances where a single cotton boll is identified as multiple ones. Additionally, due to the proximity of cotton bolls, multiple instances are detected as a single cotton boll. The second phase, occurring after the second defoliant spraying, emerges as the optimal period for cotton boll detection. During this phase, there is minimal interference from leaves, and the branching of cotton plants is less pronounced, resulting in relatively independent cotton bolls. Therefore, it is recommended to select T2 as the golden period for cotton boll detection in subsequent studies involving transfer learning. **Figure 8** illustrates the detection results of different networks on ground cotton boll images at time interval T2, with magenta boxes indicating missed detections. Despite achieving higher detection recall rates in ground cotton boll image data, the Faster R-CNN model tends to experience overfitting due to its robust deep feature extraction capabilities. This results in an increased false positive rate, significantly impacting the balance between precision and recall. The YOLO v5 model exhibits some shortcomings, with less evident features and smaller cotton bolls going unrecognized. YOLOv7 employs multi-layer modification techniques in the model, halving aspect ratios, doubling channels, and reducing downsampling. Consequently, at the same volume, YOLOv7 outperforms YOLOv5 in efficiently detecting targets with higher accuracy and faster speed. However, there are still some shadowed and concealed cotton bolls that go undetected. The YOLOv8 model provides a scaled-down version based on scaling factors, catering to the requirements of cotton boll detection scenes. Nevertheless, further improvements are needed for low-resolution small target detection. The proposed YOLO SSPD in this study evidently demonstrates high-precision cotton boll recognition at the ground scale.

**Table 2:** Model testing results for ground image datasets.

Model	Time	Precision(%)	Recall(%)	F1-Score(%)	AP_50_(%)
Faster R-CNN	T1	80.3	85.2	82.7	83.9
	**T2**	**81.6**	**86.9**	**84.2**	**83.0**
	T3	78.2	82.1	80.1	81.1
YOLOv5	T1	81.1	84.8	81.9	82.2
	**T2**	**81.7**	**83.4**	**82.5**	**83.1**
	T3	79.2	81.6	80.4	81.0
YOLOv7	T1	83.1	85.2	84.1	84.8
	**T2**	**83.8**	**85.8**	**85.0**	**85.6**
	T3	80.2	82.6	81.4	81.3
YOLOv8	T1	81.8	83.8	83.7	82.1
	**T2**	**84.6**	**86.0**	**84.3**	**82.6**
	T3	80.9	81.7	82.6	82.3
YOLO SSPD	T1	84.1	87.3	85.7	86.5
	**T2**	**85.2**	**88.9**	**87.0**	**88.1**
	T3	81.1	84.6	82.8	83.9

The values are bolded to emphasize that the best-performing models for each period consistently peaked in T2.

### 3.2 Results of UAV image cotton boll detection and transfer learning

**Figure 9:**
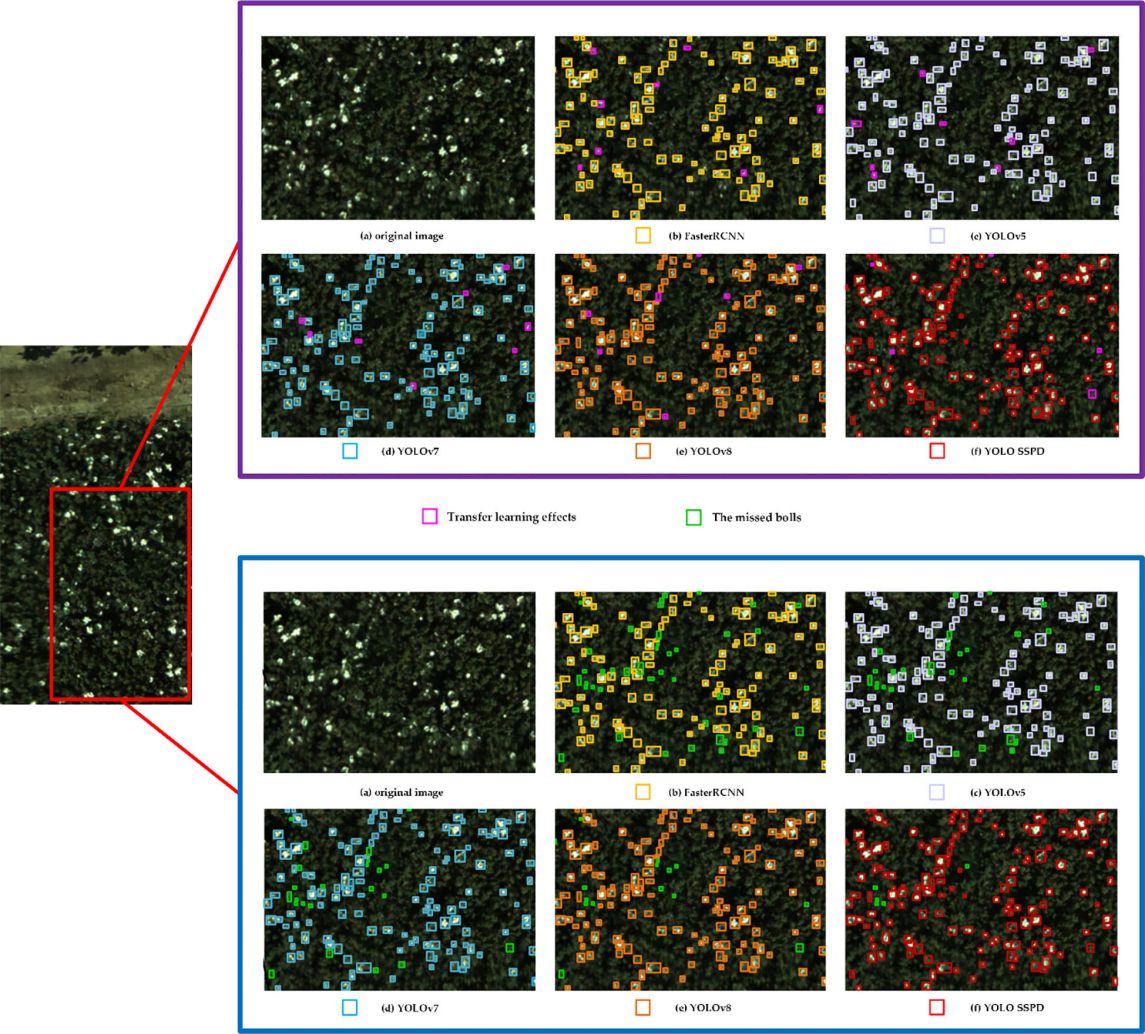
Below is a comprehensive comparison of the five object detection models before and after transfer learning. Purple boxes represent detection results before transfer learning, while blue boxes represent results after transfer learning. Different colored boxes in the images denote the effectiveness of different detection models, with yellow indicating Faster R-CNN detection, light purple for YOLOv5, blue for YOLOv7, orange for YOLOv8, and red for YOLO SSPD detection results.

The images captured by the UAV at flight altitudes of 20 meters, 40 meters, and 60 meters all exhibit distinct features of open cotton bolls, with the images obtained at a 20-meter flight altitude having the highest resolution. The contrast between the target cotton bolls and the background is more pronounced, resulting in the highest detection accuracy. Subsequent research focuses on the UAV image dataset obtained at a 20-meter altitude. When evaluating the impact of transfer learning, **Tables 3**, **4** present the cotton boll detection results using the five aforementioned detection models on the UAV RGB image dataset during the T2 period, along with the results after transfer learning on the UAV images during the same period. The detection results of different models on cotton boll images are depicted in **Figure 9**. Due to the small scale of detection targets on the drone, a portion of the region enclosed by red rectangles in the original image detection results was cropped for comparison. Comparative analysis of detection results before and after model transfer indicates overall improvement in the detection efficiency of all model’s post-transfer, with the YOLO SSPD model exhibiting the highest detection efficiency. Before model transfer, the detection time for each image in the drone RGB image dataset was 51ms, while after model transfer, the average detection time for each image in the drone RGB image dataset was reduced to 22ms. These results signify the effectiveness of model transfer. The optimal YOLO SSPD model achieves an optimal balance between detection accuracy and detection rate.

**Table 3:** UAV image datasets models testing results.

Model	Time	Precision(%)	Recall(%)	F1-Score(%)	AP_50_(%)
Faster R-CNN	T2	77.6	84.3	80.8	83.2
YOLOv5	T2	80.3	84.2	82.2	83.6
YOLOv7	T2	82.1	85.6	83.9	84.1
YOLOv8	T2	82.6	86.1	83.8	84.6
YOLO SSPD	**T2**	**85.3**	**88.0**	**86.6**	**86.9**

The bolding is used to highlight the superior metrics of the best-performing models.

**Table 4:** Testing results after models transfer.

Model	Time	Precision(%)	Recall(%)	F1-Score(%)	AP_50_(%)
Faster R-CNN	T2	79.9	85.6	82.7	83.9
YOLOv5	T2	81.1	86.4	84.8	84.3
YOLOv7	T2	83.8	87.1	85.4	86.0
YOLOv8	T2	84.1	87.2	85.6	86.4
YOLO SSPD	**T2**	**87.4**	**89.3**	**87.8**	**88.0**

The bolding is used to highlight the superior metrics of the best-performing models.

### 3.3 Validation of cotton boll detection models

**Figure 10:**
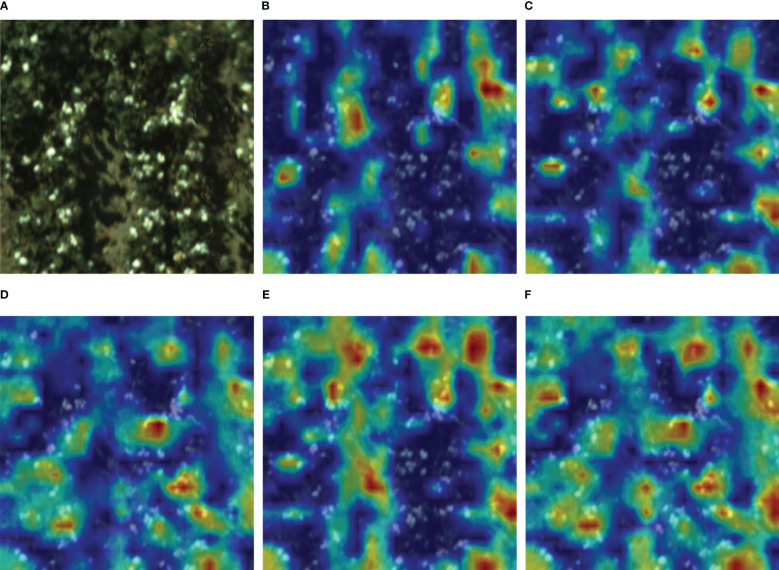
Five object detection models’ heatmaps: **(A)** Original image, **(B)** Faster R-CNN, **(C)** YOLOv5, **(D)** YOLOv7, **(E)** YOLOv8, **(F)** YOLO SSPD.

Neural networks are often perceived as black-box models with limited interpretability. However, employing class activation maps (CAM) on a trained model allows for a visual understanding of its principles. CAM (https://github.com/jacobgil/pytorch-grad-cam) typically operates on the last convolutional layer of the model to extract class activation maps corresponding to input images (Zhou et al., 2016). These CAMs, which are the same size as the input images, facilitate the visualization of predicted class scores and highlight detected objects. The generation of heatmaps involves overlaying weighted feature maps obtained from CAM. Within these heatmaps, the degree of network response in different regions of the input image can be observed. Larger heatmap ranges indicate the presence of more predicted class targets in the corresponding regions, while darker colors signify greater contributions to the predicted results. To further enhance cotton boll detection, a visual analysis of the detection results for each model was conducted through heatmap visualization, providing insights into the neural network models. As shown in **Figure 10**, Faster R-CNN focuses on prominent features of cotton bolls, making it susceptible to information loss in small target detection, evident in the discrete distribution of the heatmap. YOLOv5’s feature pyramid structure exhibits limitations in recognizing obscured and smaller cotton boll features accurately. While YOLOv7 has a larger model width and depth compared to YOLOv5, resulting in the extraction of more features, the heatmap’s predominantly light colors indicate that these positions contribute less to the network output, indicating insufficient feature extraction for practical applications. YOLOv8, with its ability to adjust the model scale for detection, outperforms the first three models in small target scenarios. However, the large-scale field images captured by the UAV exhibit diverse characteristics of open cotton bolls and suffer from lower resolution issues. This leads to YOLOv8’s focus on concentrated open cotton bolls, indicating a need for further attention to the discrete small cotton boll targets. YOLO SSPD, by introducing SPD convolution and a small target detection head onto the YOLOv8 model, significantly captures a broader target range in low-resolution small target images, achieving precise detection in the images.

### 3.4 Validation of cotton boll counting model

**Figure 11:**
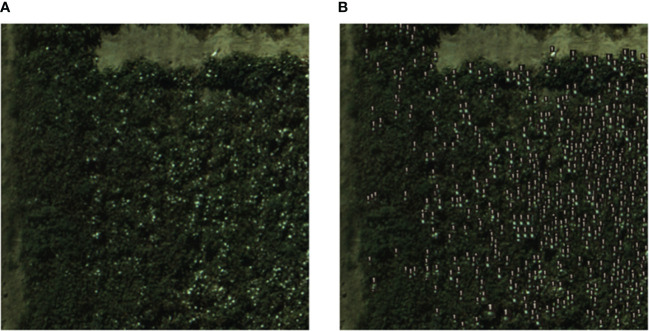
The model detection results: **(A)** Real ground boll counts, **(B)** YOLO SSPD results (UAV imagery).

This study employed the determination coefficient, RMSE, and RRMSE as metrics to evaluate the counting effectiveness of the model. Combining the YOLO SSPD detection model with transfer learning, counting was performed on UAV RGB image data. The results demonstrate that the detection model, after being fine-tuned through a transfer learning approach, achieved an R² of 0.86, RMSE of 12.38, RRMSE of 11.19%, and an AP of 88.9%, thus indicating a robust counting performance. **Figure 11** showcases how the integration of the YOLO SSPD model with transfer learning techniques enhances its ability to detect and count cotton bolls accurately in 20m resolution UAV images during the T2 period.

## 4 Discussion

Boll detection in the pre-harvest stage of cotton can realize the assessment of cotton yield, so as to provide scientific and effective resource allocation and management strategies. As cotton bolls are not obvious in the early growth stage in a complex field background environment, the stages of cotton flocculation can be selected to accurately and reliably identify and locate cotton bolls. In this study, the three stages of cotton flocculation were selected to be captured by UAV and on the ground. In order to reduce the interference of cotton leaves and achieve better detection conditions, 5 days after the first spraying of defoliant (T1), 3 days after the second spraying of defoliant (T2), and 7 days before the cotton picking (T3) were selected, and the image of T2 got the best detection accuracy in the subsequent experimental results. In the process of cotton boll data acquisition, although the effects of UAV shooting time stage, weather conditions, UAV flight speed, camera shooting angle and other factors on the quality of ground image data and remotely sensed data were taken into account, factors such as different degrees of shading and background clutter in the cotton field in the natural environment still have a significant impact on the detection accuracy (Kang et al., 2022, 2023; Li et al., 2022; Li et al., 2020). Data enhancement can balance and enrich the cotton boll image datasets, better realize the acquisition of cotton boll features, and also reduce the workload of manual labelling.

For the case of boll detection by UAV in small-scale cotton fields, which is limited in resolution and insufficient in the number of samples obtained, ground photography was conducted to obtain sufficient ground open boll data. From the perspective of transfer learning, many ground images were used to train the deep learning model. After reaching a higher accuracy, the model was transferred so that the model could achieve a good detection accuracy on UAV images with a smaller dataset. The specific steps were, on the ground cotton boll image datasets, to investigate the cotton boll detection effect of different target detection networks in different periods through comparative experiments. Then, on UAV RGB image data, the performance of different target detection networks on cotton boll detection at UAV scale and different periods were investigated through comparison and transfer learning (Meng et al., 2019). In terms of model performance, Faster R-CNN based on Region Proposal Networks could extract target cotton bolls, but the model was complex, had slow training speed, and was prone to overfitting. Due to different growth conditions, cotton bolls during the boll spitting period exhibit varying shapes and color characteristics. The feature extraction capability of Faster R-CNN was too strong, leading to failures of recognizing some cotton bolls. YOLOv5 introduced CSPDarknet53 as the backbone network and employed the PANet structure to enhance feature fusion, demonstrating good performance in both accuracy and speed. However, when applied to cotton boll detection in UAV images, the YOLOv5 model produces numerous instances of false negatives. YOLOv7 builds on YOLOv5 by introducing architectures such as the Efficient Layer Aggregation Network, but it exhibits weak generalization, with variations in different scenes and poor performance in small object detection tasks. YOLOv8 was the latest achievement in the YOLO series at the time, featuring adjustable scaling coefficients and excellent application in practical scenarios with small targets. The proposed YOLO SSPD object detection model further improves the detection accuracy of small cotton bolls from UAVs by building upon YOLOv8. Experimental results indicate that YOLO SSPD performs best on both the ground cotton boll image dataset (T2) and the UAV RGB image dataset(T2). The accuracy of cotton boll detection in UAV scale is enhanced through the transfer model, contributing to improved accuracy in cotton yield prediction (Wang et al., 2021; Rodriguez-Sanchez et al., 2022). The combination of the YOLO SSPD detection model and transfer learning methods excels in detecting cotton bolls in complex environments from UAV RGB image data, providing a more precise representation of the specific locations of targets. The counting results accurately reflect the number of cotton bolls during the boll spitting stage, closely matching actual counting results (Siegfried et al., 2023). Utilizing the YOLO SSPD model for counting cotton bolls in UAV-scale images can be appropriately applied in practical cotton production processes (Qiu et al., 2022; Lang et al., 2023).

Although some progress has been made in this study, there are still many issues that need to be explored and solved in depth. (1) This study is based on cotton boll image datasets collected by ground and UAV at three altitudes (20 m, 40 m and 60 m). The image resolution of the images collected at 40 m and 60 m flight altitudes is not high, which impacts the precision of cotton boll detection and recognition. The UAV can be upgraded subsequently in terms of the camera pixels and the frame rate. High-resolution UAV images are able to achieve higher accuracy using the method proposed in this paper. (2) In the future, with a focus on enhancing the efficacy of cotton boll detection, multi-scale image fusion algorithms can be targeted to expand the detection area while improving the image resolution. Further, the large-scale cotton field yield estimation combined with satellite remote sensing images can be practically applied to a broader range of production research.

## 5 Conclusions

This study proposes a target detection network, YOLO SSPD, based on YOLOv8, specifically designed for detecting cotton bolls during the boll spitting period. In ground-based cotton boll image detection, the model was trained alongside four other object detection models until convergence. Subsequently, transfer learning was employed to apply these models to UAV-based cotton boll image detection. A comparison with four other models shows that YOLO SSPD outperforms them all. In the T2 period, the detection accuracy on UAV cotton boll images reaches 0.874, and the cotton boll count R² is 0.86. The results indicate that utilizing transfer learning and the YOLO SSPD detection model significantly improves the accuracy of cotton boll detection. The outcomes of this study serve as a practical tool in the cotton production process, enhancing the efficiency of cotton information detection. They also provide a basis for agricultural researchers to make timely decisions in cotton management, ultimately improving cotton yield and quality.

## Data availability statement

The original contributions presented in the study are included in the article/supplementary material. Further inquiries can be directed to the corresponding authors.

## Author contributions

MZ: Conceptualization, Investigation, Methodology, Writing – original draft. WC: Conceptualization, Resources, Software, Writing – review & editing. PG: Conceptualization, Funding acquisition, Project administration, Supervision, Writing – review & editing. YL: Methodology, Writing – review & editing. FT: Validation, Visualization, Writing – review & editing. YZ: Data curation, Validation, Writing – review & editing. SR: Validation, Writing – review & editing. PX: Formal analysis, Writing – review & editing. LG: Data curation, Validation, Writing – review & editing.
